# Remineralization of Artificial Dentin Caries Using Dentin and Enamel Matrix Proteins

**DOI:** 10.3390/ma12132116

**Published:** 2019-07-01

**Authors:** Katja Bächli, Patrick R. Schmidlin, Florian Wegehaupt, Frank Paqué, Liza Ramenzoni, Sander Botter

**Affiliations:** 1 Clinic of Conservative and Preventive Dentistry, Center of Dental Medicine, University of Zurich, 8032 Zurich, Switzerland; 2 Balgrist Campus AG, 8008 Zürich, Switzerland

**Keywords:** tooth remineralization, dentin, micro-CT, DMP, EMD

## Abstract

To assess the remineralizing potential of dentin matrix proteins and enamel matrix derivatives (DMPs and EMDs) after application on artificially induced dentin lesions, given the hypothesis that these materials increase the mineral uptake, binding, and mineralization. Forty-eight caries-free human premolars were used. Teeth were cut, polished, and embedded, leaving an open window on the root surface, of which one-third was covered with a flowable composite to preserve the healthy untreated dentin. Then, samples were demineralized in Buskes solution for 33 days. A micro-CT scan prior to treatment was performed. Next, the samples were randomly allocated into four groups: (A) An untreated negative control (CON), (B) application of porcine dentin matrix proteins (DMP), (C) treatment with enamel matrix derivatives (EMD, Emdogain, Straumann), and (D) amine fluoride application (AMF, Elmex fluid, GABA). All samples were placed in artificial saliva for 21 days. A second micro-CT scan was performed, after which the change in gray scaling within a defined region of interest (0.25 mm^3^) was analyzed. ANCOVA was applied to discover statistical differences between the different treatments. Both, treatment with AMF; (P = 0.011 versus CON) as well as with DMP (P = 0.043 versus CON) yielded a statistically significant difference compared to the control treatment. EMD treatment was not found to differ (P > 0.05). Mainly the top layer of the defects showed clear signs of remineralization, which was also evident in CON. This study was able to visually confirm the remineralization potential of demineralized dentin especially after DMP application, which, however, did not outperform AMF. Based on this, additional studies combining proteins and fluorides are now warranted and ongoing.

## 1. Introduction

Prophylaxis and treatment of root caries is becoming increasingly important. As the number of elderly people is growing, the time span in which their teeth are used becomes longer. [1]. Dentin consists of approximately 70% inorganic matrix, 20% organic matrix, and 10% water by weight [2]. The inorganic part consists of apatite crystals (Ca_10_(PO_4_)_6_(OH)_2_) [3]. The organic part contains approximately 90% type I collagen and about 10% noncollagenous proteins (NCPs) [4]. Macrostructurally, it is transversed by tubules, which are oriented radially from the pulp to the dentin–enamel junction and surrounded by a highly mineralized cuff containing the apatite crystals [5] together with a small amount of collagen [6], known as the peritubular dentin [5]. The interstitial space between the peritubular cuffs is called the intertubular dentin and it consists of collagen fibrils that are bound by crystalline apatite [5]. Odontoblasts generate immature predentin as a template for the maturation process, which is characterized by the deposition of apatite crystals in the fibrillar matrix and a subsequent mineralization process, leading to the formation of mature dentin [7]. This process is controlled mainly by noncollagenous proteins (NCPs) [8]. Among these, dentin matrix proteins (DMPs) are predominantly found in the extracellular matrix of the dentin [8]. This group of proteins includes dentin matrix protein 1 (DMP1), dentin phosphophoryn (DPP) or dentin matrix protein 2 (DMP2), dentin sialoprotein (DSP), and DMP4 [8]. The DMPs display calcium-binding properties controlling and leading to distinct tissue calcification patterns [8]. These properties make DMPs attractive candidates for dentin remineralization and regeneration strategies [8].

Whenever the root becomes exposed to the oral cavity after recessions have formed, the structural long-term integrity is influenced by a plethora of mechanical and chemical attacks, but also by prophylactic countermeasures (e.g., the use of fluorides) [9]. An imbalance between de- and remineralization favouring a net mineral loss can result in caries lesion formation or erosion [10]. At the start of lesion formation, the minerals are released from the outer surface by a fine gradient, whilst preserving the collagen fibers [11]. Later on, the collagen fibers are broken down by proteolytic enzymes from colonizing bacteria, thereby losing their structural properties [11]. Whereas removing mineral ions from hydroxyapatite (HA) crystals leads to demineralization, remineralization processes add or substitute lost mineral ions to the HA crystals [3]. However, the remineralization of dentin as a complex native structure is demanding, because of the lack of apatite seed crystallites along the lesion surface for crystal growth [10]. 

Since specialized native tooth proteins or derivatives, to which the apatite crystals can bind, play a key role in the physiologic process of remineralization, this study aimed to apply dentin matrix proteins (DMPs) and enamel matrix derivates (EMDs) on artificially induced dentin lesions and to assess their remineralization potential. The role of EMDs, which are commercially available as a product mainly used for guided tissue regeneration [12], has already been studied in the remineralization process of enamel, but only a few attempts have been made to evaluate its potential in remineralization processes of dentin [13]. Data on the effects of DMPs in remineralization are still scarce.

In this study, we therefore simulated and quantified the remineralization potential of both protein classes. Their effects were compared against fluoride, which can be considered as a powerful remineralization agent and is responsible for an overall significant decline in caries prevalence worldwide [14], thus, serving as a positive control in this study. The null hypothesis was that all treatment modalities would cause an equal increase in mineral uptake and mineralization.

## 2. Materials and Methods

### 2.1. Isolation of DMPs According to Schlagenhauf

The Isolation of the DMPs was performed according to Schlagenhauf/Augello. [15,16] Caries-free porcine teeth from fresh upper jaws obtained from a local slaughterhouse were extracted and the roots were cleaned with curettage. The crown and the root were separated with an IsoMet Low Speed Saw (Buehler, Düsseldorf, Germany) and the roots were cut into 1.5–2 mm thick slices. Throughout the experiment, samples were stored at 5 °C in tap water. To degrease the slices, samples were put in 50 mL ethanol and 50 mL chloroform for 10 min, three consecutive times. Afterward, the slices were shredded with the aid of nitrogen and ground with a mill (MM 400, Retsch, Hahn, Germany) in a zircon grinding breaker with a zircon ball for 4 min at 27 hertz. From the resulting powder, 2.5 g was dissolved in 50 mL 0.5 M HCl. After 24 h, the solution was centrifuged at 4000 rpm for 10 minutes. The supernatant was removed, and the remaining part was dissolved again in 50 mL 0.5 M HCl. This process was repeated four times. The remaining final part was dissolved in 50 mL 4 M guanidine-HCl solution for 96 h under constant stirring.

For dialysis, the tubes were boiled for 10 min in a solution of 1.1 g ethylenediaminetetraacetic acid disodium salt and 1.2 g sodium carbonate dissolved in 600 mL deionized water. After cooling down, they were stored in a 5 mM EDTA solution.

The solution of the milled teeth in 4 M guanidine-HCl was filled into dialysis tubes, which were placed into 500 mL of deionized water. The deionized water was changed daily for three days. Finally, the content of the dialysis tubes was lyophilized for 48 h (ALPHA 2-4, Christ, Osterode am Harz, Germany).

### 2.2. Sample Preparation

**Figure 1:**
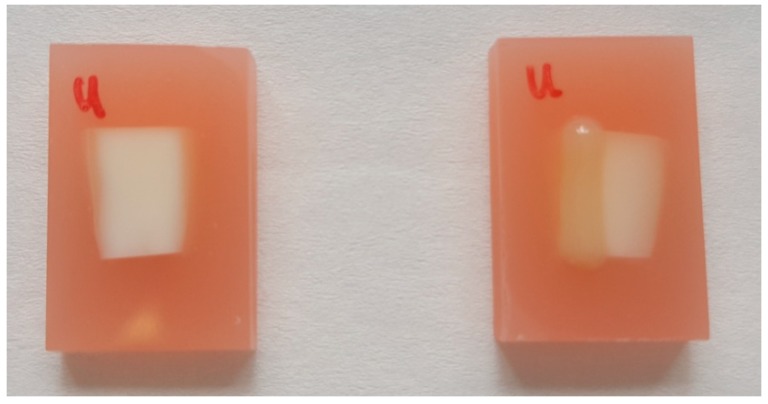
Human premolars were cut and the roots polished and embedded in PMMA, leaving an exposed surface area of approximately 4 × 8 mm^2^. On the right-hand side, a sample is shown in which one-third of the available surface has been covered with a flowable composite to preserve the healthy untreated dentin.

Forty-eight, caries-free, lower human premolars were used as test samples. The premolars were collected at the Center of Dental Medicine of the University of Zürich as a by-product of planned treatment. The use of the teeth in this study had no influence on the treatment of the patients. The teeth were collected anonymously. The patients agreed in writing for the use of the teeth for scientific purposes. Following local legal regulations, no ethic approval had to be obtained in the case of research with irreversible anonymized biological materials. The crown was cut 1 mm apical of the cervical line. The root was cleaned with curettage. The apex was shortened, leaving a uniform sample length of 8 mm. Samples were then embedded in PMMA (Paladur-Rosa, Heraeus Kulzer, Hanau, Germany). The surface was polished (PLANPOL 2, Struers, Ballerup, Denmark) with P1200, P2500, and finally P4000 grinding paper. The protruding exposed surface area had a size of approximately 4 × 8 mm^2^. One-third of this area was covered with a flowable composite resin material (Filtek Supreme XTE Flow, 3M ESPE, Seefeld, Germany), as shown in Figure 1.

The demineralization acidic buffer solution was prepared according to Buskes [17] by mixing 5 L distilled water, 2.205 g CaCl_2_ + 2H_2_O (3.0 mM), 2.041 g KH_2_PO_4_ (3.0 mM), 10 mL MHDP solution (prepared from 100 mL distilled water with 0.0528 g methylene diphosphoric acid), 14.3 mL CH_3_COOH (50 mM), and 10 M KOH to titrate the solution to pH 4.95. The samples were stored in this demineralizing solution at 37 °C for 33 days. The solution was changed every two to three days. After demineralization, another third of the exposed dentine window was covered with the flowable material in order to protect approximately one-half of the demineralized area. 

Finally, samples were randomly divided into four groups by a blinded examiner (P.R.S.).

### 2.3. Sample Treatment

Each group consisted of 12 samples. Prior to treatment, all samples were dried for three seconds with oil-free compressed air. Immediately after this, fifty microliters of each corresponding solution (see below) were pipetted on the exposed demineralized dentine surface, without touching the surface, and left in situ for 5 min: 

(A) Control (CON)

The samples remained untreated and served as control. After the drying, the samples were placed immediately in artificial saliva.

(B) Dentin matrix proteins (DMPs)

Lyophilized DMPs were dissolved in 1 mL 0.05 M acetic acid and stored for 30 min at 4 °C to a final concentration of 30 mg/mL before use. The treatment occurred as explained above.

(C) Enamel matrix derivatives (EMDs; Emdogain, Straumann):

Lyophilized EMDs were also dissolved in 1 mL 0.05 M acetic acid and stored for 30 min at 4 °C to a final concentration of 30 mg/mL before use. The treatment procedure was analogous to that of group (B).

(D) Amine fluoride solution (AMF):

Amine fluoride solution was applied (Elmex fluid, GABA, Therwil, Switzerland) as supplied by the manufacturer analogous to groups (B) and (C).

Afterward, the remaining excess solutions were removed using a paper towel. All samples were then immediately transferred into 20 mL of artificial saliva [13] (KCl 2.4 g, NaCl 1.7 g, MgCl_2_·6H_2_O 0.1 g, CaCl_2_·2H_2_O 0.2 g, KSCN 0.2 g, KH_2_PO_4_ 0.7 g, H_3_BO_3_ 0.1 g, and aqua dest. ad 1 L) for 24 h at 37 °C. Afterward, each sample was again transferred into 10 mL of fresh artificial saliva for another 20 days at 37 °C. The storage medium was changed daily.

Unfortunately, during incubation in the artificial saliva, we noticed that the protective composite seal had detached in seven out of 48 samples, i.e., two specimens in CON, AMF, and DMP groups, and one sample in the EMD group, respectively. To rule out differences in the measurement data due to a difference in sample consistency, these samples were excluded from all analyses. 

### 2.4. Micro-CT Scanning

Samples were scanned in a micro-CT device (model: Skyscan 1176 in vivo micro-CT, Bruker/Skyscan, Kontich, Belgium) in order to visualize and measure the remineralization potential after treatment. As a noninvasive imaging instrument, this approach allows the imaging of internal structures in three dimensions, with high spatial resolution and without destroying the sample [18].

**Figure 2:**
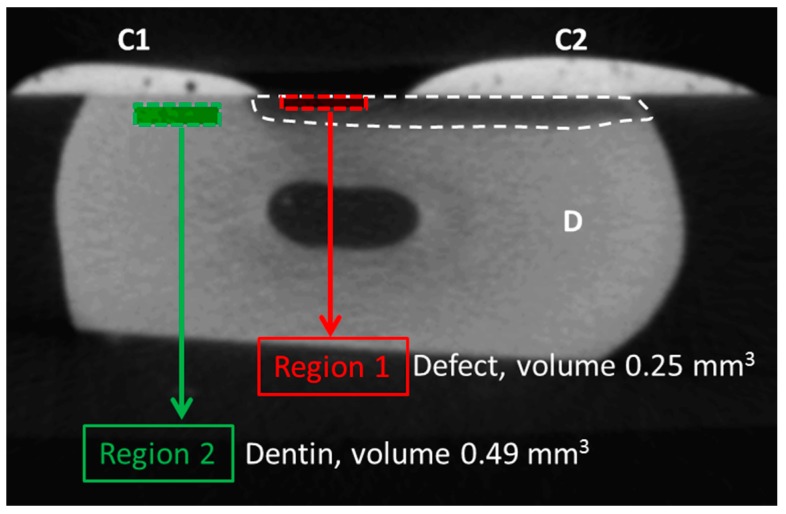
Selection of the region of interest. In each sample, composite (C1) was used to cover part of the dentin defect, after which a defect (hatched, darkened area) was created using Buskes solution. Part of the defect was then covered by composite (C2), samples were scanned with a micro-CT for the first time, after which either control treatment or treatment with amine fluoride (AMF), enamel matrix derivatives (EMDs), or dentin matrix proteins (DMPs) was initiated. Following a second round of scanning and scan alignment, two regions of interests were created. Region 1 had a volume of 0.25 mm^3^ and was positioned within the top layer of the defect created by the Buskes solution. Region 2 had a volume of 0.49 mm^3^ and was selected as a background control, to rule out technical changes, e.g., due to the scanning procedure. The ratio of the two gray scale values of both regions was taken, i.e., (region 1/region 2) × 100%. This procedure was performed within the scans made before treatment and within the scans made after treatment (i.e., control (CON), AMF, EMP, and DMP groups) and subsequent incubation in artificial saliva.

Each sample was scanned twice; once after defect induction but before treatment, and a second time following treatment and incubation in the artificial saliva for 21 days as described above. Scan settings were as follows: 17 µm voxel size, 50 kV, 500 µA, 0.5° rotation angle; 360° scan, 0.5 mm aluminum filter, 250 ms exposure/integration time, frame averaging of 3. The total scan time per sample was approximately 21 min. All scans were reconstructed into eight-bit gray scale images using NRecon v.1.7.1.0 (Bruker/Skyscan) and afterward imported into Analyze 12.0 (AnalyzeDirect, Rochester, MN, USA). Voxel depth, voxel height, and voxel width were set to 0.017 mm. Pre- and post-treatment scans were aligned using the oblique sections tools and thereafter registered using the 3D voxel registration tool. Next, originating from the exact center of each sample, two regions of interest were selected (Figure 2). Region 1 (length 200 cross sections or 3.4 mm along the tooth surface; volume 0.25 mm^3^) was located within the top layer of the defect. This region was used to measure differences between both scans due to the treatment. Region 2 (length 3.4 mm; volume 0.49 mm^3^) was located deeper within the healthy dentin. The function of region 2 was to act as an internal control and to rule out differences in the gray scaling of the tissue between both pre-and post-treatment scans. The average gray scale of both regions was calculated within the pre- and post-treatment scans and was then expressed as follows: ((gray scale region 1)/(gray scale region 2)) × 100%.

To further quantify the amount of mineral deposited due to treatment, the mineral-containing voxels with a gray scale of at least 35 (of maximum 255) within region 1 were segmented using a global threshold strategy. 

Before and after treatment, samples were scanned in batches consisting of 11 randomly selected samples; the 11th sample of each batch always consisted of one and the same sample derived from the CON group (Sample ID #41) and acted as a control reflecting the technical variation of the scan procedure; after comparing the average gray scale values of this sample between scan batches, the average difference between scan batches was shown to be 2.4% (data not shown).

### 2.5. Data Analysis and Statistics

Figures were prepared in GraphPad Prism 7.04 (GraphPad Software, San Diego, CA, USA). For statistical analysis, an ANCOVA was performed in SPSS Statistics 23 (IBM, Armonk, NY, USA). Prior to analysis, an ANOVA was performed on the pre-treatment data to confirm that the observed pre-treatment differences between control and treatment groups were nonsignificant; likewise, the homogeneity of regression was checked. Both tests gave nonsignificant results, indicating the validity of the ANCOVA use.

## 3. Results

**Figure 3:**
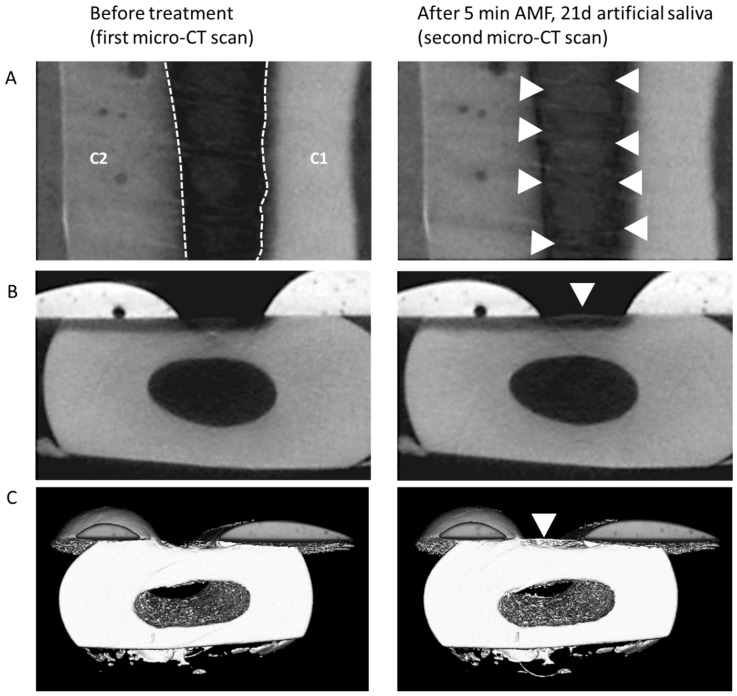
(**A**) Example of mineralization differences in a sample before treatment and following the 5 min treatment (shown: AMF) and incubation in artificial saliva for 21 days. Top view of gray scale images; C1/C2: protective composite material (see caption of Figure 4); hatched borders indicate the area of the defect, appearing relatively dark due to the absence of minerals. After treatment (right panel), an increase in mineralization was observed (arrow heads). (**B**) Side view of the same sample. (**C**) 3D-reconstruction of registered and segmented datasets of the same sample, with the increase in mineral located within the defect (arrowhead).

**Figure 4:**
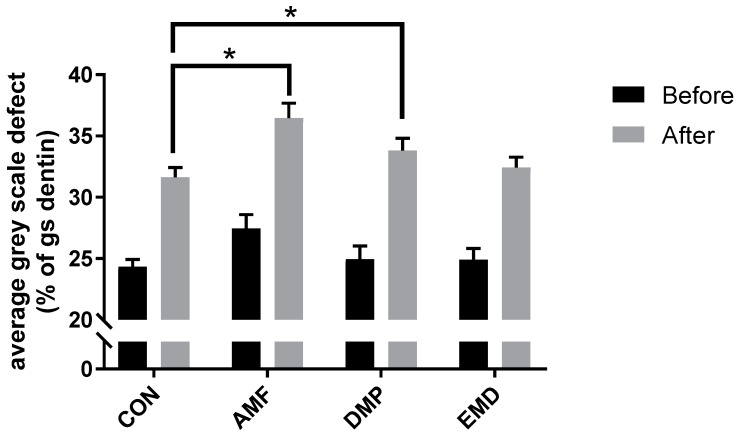
Quantification of gray scale differences as measured in “region 1” within the top layer of the defect before and after treatment. Differences were expressed as percentage of the average gray scale measured in “region 2” located deeper within the healthy dentin. CON—control; AMF—amine fluoride; DMP—dentin matrix proteins; EMD—enamel matrix derivatives, shown are averages ± SEM, * P < 0.05.

**Figure 5:**
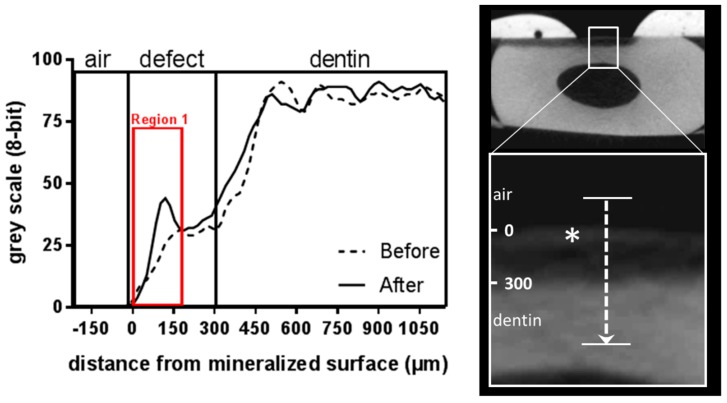
Gray scale intensity profile measured along a longitudinal axis within “region 1” before and after treatment (shown: EMD). On the right hand, the corresponding gray scale image after treatment is shown. In both the intensity profiles as in the gray scale image the “crust” within the defect is highlighted with an asterisk.

Using a simple one-point line measurement, the depth of the defects prior to treatment was measured to be between 260 and 280 µm deep in each treatment group. After the 5 min treatment, the defect remineralized (Figure 3), although this effect was observed within all four analyzed groups, i.e., also in the control group (Figure 4; Figure S1; difference between black and gray bars). Nonetheless, we found that both, treatment with AMF (P = 0.011 versus CON) as well as with DMP (P = 0.043 versus CON) yielded a statistically significant difference compared to the control treatment. Typically, the remineralization pattern in all four groups showed a crust-like superficial remineralization pattern in the top layer of the defect (Figure 5), whereas the collagen matrix layers beyond this layer remained demineralized. 

Absolute gray scale averages of “region 2” did not differ between the pre- and post-treatment scans (average ± SD; CON, before: 101.2 ± 2.8, after: 101.5 ± 2.9; AMF, before: 100.1 ± 2.6, after: 100.8 ± 2.6; DMP, before: 102.2 ± 3.5, after: 102.6 ± 3.5; EMD, before; 103.9 ± 2.9, after: 103.6 ± 2.9), confirming on the one hand the uniformity of the dentin density across groups, and at the same time indicating that the treatment procedure as well as the three-week artificial saliva incubation did not influence mineralization levels beyond the level of the defect.

## 4. Discussion

In this study, DMP and AMF both induced a significant added increase in the uptake of minerals in a model of dentin caries. In contrast, in our hands EMD treatment was not found to differ compared to the control group (CON). It was noted that mainly the top layer of the defect had been mineralized and that significant remineralization during the three-week incubation period had also taken place in the CON.

This study had some limitations. The human premolars that were used were of unknown origin; age, sex, or the date of extraction and how long they were stored had not been recorded. This may explain some of the (nonsignificant) differences at baseline between the different groups. To evaluate the remineralization, micro-CT scans were performed. Currently, transverse microradiography is considered as the gold standard for the determination of mineral loss [18]. However, this determination method requires physically cutting the specimen [18]. In contrast, micro-CT imaging serves as a noninvasive imaging tool. It allows illustrating the inner structures with high spatial resolution in three dimensions without destruction of the specimen [18] and is sensitive enough to detect a change between demineralization and remineralization [19]. Nakata et al. were able to show a strong linear correlation between the CT density and mineral content [20]. In this study, we observed an increase in mineral content in the specimen. However, it remains unclear whether crystallization of these minerals occurs and how this will affect the mechanical properties of the surface. The analysis of Magalhães et al. showed a medium but nonlinear correlation and variable relationship between mineral content and cross-sectional hardness [21]. Therefore, the findings of this study do not yet justify assumptions on native (i.e., anatomical–functional) regeneration of the dentin. On the other hand, our results are in line with other studies that also observed an effect of DMPs on demineralized dentin, supporting the conclusion that DMPs promote the deposition and nucleation of HA. For example, Bedran-Russo et al. showed in their study that “DMP1-treated samples promoted deposition of amorphous calcium phosphate (ACP) precursors and needle-shaped hydroxyapatite crystals surrounding collagen fibrils” [22]. Liu et al. came to the conclusion that “synthetic peptides derived from DMP1 bind type I collagen and promote nucleation of HA” [23]. In this in vitro study, the influence of the tooth pulp was neglected. In a dentine carious lesion, dentin matrix proteins are released and reactivated from the dentin matrix. They regulate an immune response involving dental pulp stem cell differentiation and, thus, play a noteworthy role during dental pulp repair [24]. Finally, during sample preparation and treatment, the surface of the samples had to be dried. Once exposed, the collagen fibers are very fragile and may have been damaged during this process. Furthermore, we generated relatively deep lesions, so we may assume that within these lesions, the collagen fibers partly collapsed. Thanatvarakorn et al. showed that the collagen network acts as a natural barrier [25]; in vivo, the collagen fibers are broken down by proteolytic enzymes from the invading/colonizing bacteria and lose their structural characteristics [11]. In this in vitro study, the influence of the proteolytic enzymes from the colonizing bacteria was not included. However, it is assumed that there is no influence on lesion remineralization in vitro when removing demineralized collagen by enzymes [25].

Within the limitations of this study, it might be concluded that the application of DMPs on artificial carious dentin lesions can improve the mineralization of these lesions, albeit mainly within the top layer of the demineralized collagen network. 

The DMPs offer a simple treatment of dental lesions. In this study, the DMPs were applied to the surface of the specimen without rubbing the detergent. Further examination is necessary to determine the influence of the way the detergent is applied to the lesion of the dentine, with respect to the alignment of collagen fibers as discussed above. Further studies are needed to verify this approach, also under pH-cycling conditions and in in vivo models. The DMPs were extracted out of porcine teeth, analogous to EMD. Porcine teeth are easily accessible and allow large-scale commercial production of DMPs. Interestingly, it is also possible to extract DMPs out of human teeth, for example after the extraction of the third molar, which can then be used for later use within the same individual. Whether this approach achieves an even greater potential for remineralization should be investigated.

## 5. Conclusions

This study was able to confirm the remineralization potential of DMPs at least to the same extent as AMF. More studies, especially combining proteins and fluoride are now warranted.

## Supplementary Materials

The following are available online at https://www.mdpi.com/1996-1944/12/13/2116/s1, Figure S1: Quantification of the amount of mineral within “region 1” following global thresholding before and after treatment. Only voxels with a minimum gray scale of 35 (out of maximum 255) were counted and results were expressed as the percentage of region 1 (0.25 mm^3^) filled with such voxels. CON—control; AMF—amine fluoride; DMP—dentin matrix proteins; EMD—enamel-matrix derivatives, shown are averages ± SEM.
