# Supplementary material for: Remineralization of Artificial Dentin Caries Using Dentin and Enamel Matrix Proteins
Source: Materials (Basel). 2019 Jul 1;12(13):2116. doi: 10.3390/ma12132116 (PMC6651374; doi:10.3390/ma12132116)
Supplement: Supplementary file 1 [file materials-12-02116-s001.pdf]

materials

Article

Remineralization of Artificial Dentin Caries Using Dentin and Enamel Matrix Proteins

Katja Bächli [1], Patrick R. Schmidlin [1,*], Florian Wegehaupt [1], Frank Paqué [1], Liza Ramenzoni [1] and Sander Botter [2]

[1] Clinic of Preventive Dentistry, Periodontology and Cariology, Center of Dental Medicine, University of Zurich, Zurich 8032, Switzerland; katja.zobrist@hotmail.com (K.B.); florian.wegehaupt@zzm.uzh.ch (F.W.); frank.paque@zzm.uzh.ch (F.P.); liza.ramenzoni@zzm.uzh.ch (L.R.)

[2] Balgrist Campus AG, Zürich 8008, Switzerland; sander.botter@balgristcampus.ch

* Correspondence: patrick.schmidlin@zzm.uzh.ch; Tel.: +41-(0)44-634-32-84

Received: 11 June 2019; Accepted: 29 June 2019; Published: 1 July 2019

Supplementary Materials:

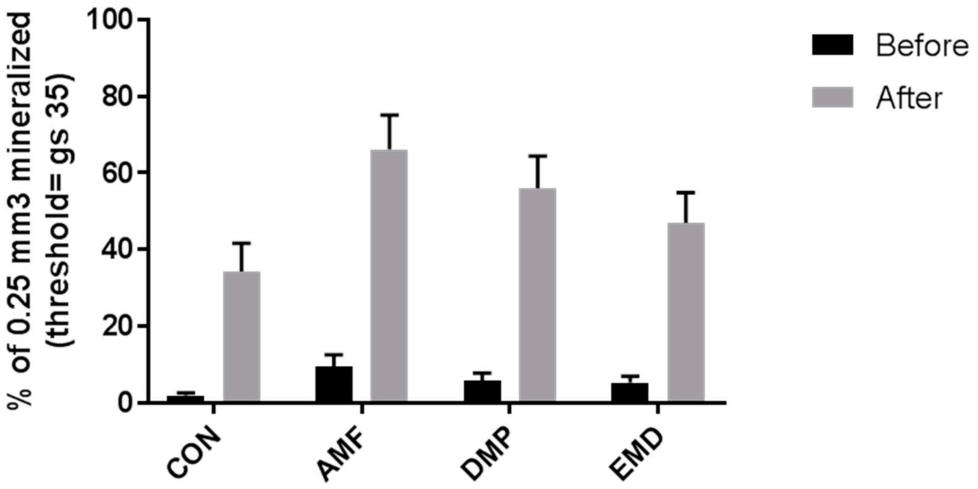

Figure S1. Quantification of the amount of mineral within "region 1" following global thresholding before and after treatment. Only voxels with a minimum grey scale of 35 (out of maximum 255) were counted and results were expressed as the percentage of region 1 (0.25 mm^3) filled with such voxels. CON = control; AMF; amine fluoride; DMP = dentin matrix proteins; EMD = enamel-matrix derivatives, shown are averages +/− SEM.

© 2019 by the authors. Submitted for possible open access publication under the terms and conditions of the Creative Commons Attribution (CC BY) license (http://creativecommons.org/licenses/by/4.0/).
